# Finding Cures for Tropical Diseases: Is Open Source an Answer?

**DOI:** 10.1371/journal.pmed.0010056

**Published:** 2004-12-28

**Authors:** Stephen M Maurer, Arti Rai, Andrej Sali

## Abstract

The Tropical Disease Initiative will be a Web-based, community- wide effort where scientists from the public and private sectors join together to discover new treatments

Only about 1% of newly developed drugs are for tropical diseases, such as African sleeping sickness, dengue fever, and leishmaniasis [1]. While patent incentives and commercial pharmaceutical houses have made Western health care the envy of the world, the commercial model only works if companies can sell enough patented products to cover their research and development (R&D) costs. The model fails in the developing world, where few patients can afford to pay patented prices for drugs.

It is easy and correct to say that Western governments could solve this problem by paying existing institutions to focus on cures for tropical diseases. But sadly, there does not appear to be enough political will for this to happen. In any case, grants and patent incentives were never designed with tropical diseases in mind.

Two main kinds of proposals have been suggested for tackling the problem. The first is to ask sponsors—governments and charities—to subsidize developing-country purchases at a guaranteed price [2,3,4]. The second involves charities creating nonprofit venture-capital firms (“Virtual Pharmas”), which look for promising drug candidates and then push drug development through contracts with corporate partners. In this article, we discuss the limitations of these two approaches and suggest a third, “open source,” approach to drug development, called the Tropical Diseases Initiative (TDI). We envisage TDI as a decentralized, Web-based, community-wide effort where scientists from laboratories, universities, institutes, and corporations could work together for a common cause (see www.tropicaldisease.org).

## Why Open Source?

The idea behind asking sponsors to subsidize developing country purchases at a guaranteed price is that this will prop up drug prices and restore incentives for developing new drugs [2,3,4]. In other words, it is a way of fixing the patent problem. However, subsidies have an important weakness: it is almost impossible to determine correctly how large the subsidy should be. In principle, the most cost-effective solution is to set a subsidy that just covers expected R&D costs. But how large is that? R&D costs are very poorly known, with the published estimates quoting uncertainties exceeding $100 to $500 million per drug. If the subsidy is set too low, companies cannot cover their R&D costs and nothing will happen. Set the subsidy too high, and the sponsor's costs skyrocket. To date, no sponsor has tried to implement these proposals.

In the “Virtual Pharma” approach, governments and philanthropies fund organizations that identify and help support the most promising private and academic research. Examples include the Institute for One World Health (www.iowh.org), a not-for-profit pharmaceutical company funded mainly through private sources and the Gates Foundation, and the Drugs for Neglected Diseases Initiative (www.dndi.org), a public sector not-for-profit organization designed to mobilize resources for R&D on new drugs for neglected diseases.

Virtual Pharmas have clearly started to bear fruit, and are responsible for most candidate treatments for tropical diseases currently under development. For example, the Drugs for Neglected Diseases Initiative has a portfolio of nine projects spread out across the drug development pipeline for the treatment of leishmaniasis, sleeping sickness, Chagas disease, and malaria [6]. But Virtual Pharmas face three important problems. The first is similar to the problem faced by subsidy proposals: guessing private-sector R&D costs. One needs to understand what a product costs in order to negotiate the best possible price—and guessing wrong is likely to be expensive. Second, Virtual Pharma's development pipelines will run dry without more upstream research. Research has been particularly weak in exploiting genomic insights [7]. Third, tropical disease research is badly underfunded. For this reason, Virtual Pharma cannot succeed without rigid cost containment.

We believe that a new, community-wide consortium, the Tropical Disease Initiative, can help solve these problems. Its success would help keep Virtual Pharma's R&D pipeline full. Furthermore, it would use open-source licenses to keep its discoveries freely available to researchers and—eventually—manufacturers. As we explain below, well-designed open-source licenses are the key to containing Virtual Pharmas' R&D costs.

While we expect the final choice of license to be made by TDI's members, the guiding principle should be to pick whatever license lets developing country patients derive the most benefit from TDI's work. Possible choices are shown in Box 1.

**Box 1. Possible Licenses for TDI Discoveries**

- A public-domain license that permits anyone to use the information for any purpose.
- Licenses similar to the Creative Commons Attribution License (http://creativecommons.org/licenses/by/2.0) that permit anyone to use the information for any purpose, provided proper attribution is given.
- Licenses such as the General Public License (www.opensource.org/licenses/gpl-license.php) that prohibit users from seeking intellectual property rights.
- Licenses that permit commercial companies to obtain and exploit patents outside the developing world. These would allow Virtual Pharma to stretch its own R&D funds by letting corporate partners sell patented products to ecotourists, governments, and other consumers living in the industrialized world.

## How It Works

To date, open-source methods have made little headway beyond software [8]. However, computing and computational biology are converging. In the same way that programmers find bugs and write patches, biologists look for proteins (“targets”) and select chemicals (“drug candidates”) that bind to them and affect their behavior in desirable ways. In both cases, research consists of finding and fixing tiny problems hidden in an ocean of code.

**Figure 1:**
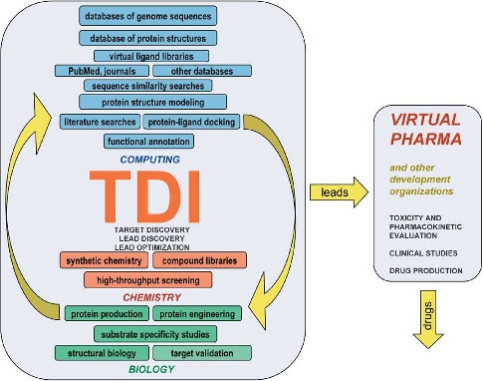
The TDI Model of Online Collaboration

What would open-source drug discovery look like? As with current software collaborations, we propose a Web site where volunteers use a variety of computer programs, databases, and computing hardware (Figure 1). Individual pages would host tasks like searching for new protein targets, finding chemicals to attack known targets, and posting data from related chemistry and biology experiments. Volunteers could use chat rooms and bulletin boards to announce discoveries and debate future research directions. Over time, the most dedicated and proficient volunteers would become leaders.

Ten years ago, TDI would not have been feasible. The difference today is the vastly greater size and variety of chemical, biological, and medical databases; new software; and more powerful computers. Researchers can now identify promising protein targets and small sets of chemicals, including good lead compounds, using computation alone. For example, a SARS protein similar to mRNA cap-1 methyltransferases—a class of proteins with available inhibitors—was recently identified by scanning proteins encoded by the SARS genome against proteins of known structure [9]. This discovery provides an important new target for future experimental validation and iterative lead optimization. More generally, existing projects such as the University of California at San Francisco's Tropical Disease Research Unit (San Francisco, California, United States) show that even relatively modest computing, chemistry, and biology resources can deliver compounds suitable for clinical trials [10]. Increases in computing power and improved computational tools will make these methods even more powerful in the future.

Just as they do today, Virtual Pharmas would choose the best candidates. The difference is that open-source drugs could not be patented in developing countries. This would not stop Virtual Pharma from developing promising discoveries. (S. Nwaka, V. Hale, personal communications). Importantly, TDI would be a great boost to the efforts of Virtual Pharmas, because it would help to contain the costs of discovering, developing, and manufacturing drugs.

## Cost Containment

TDI would contain costs in three important ways. First, TDI would ask volunteers to donate their time (and any patentable discoveries) to the collaboration. Instead of financial incentives, TDI would offer volunteers non-monetary rewards, such as ideological satisfaction, the acquisition of new skills, enhancement of professional reputation, and the ability to advertise one's skills to potential employers. Software collaborations have demonstrated that these incentives are a good way to attract and motivate programmers [11]. Similar incentives should work equally well for biologists, chemists, and other scientists.

Second, we have already pointed out that existing proposals have difficulty containing costs. The root cause is patents. Normally, society relies on competition to keep prices low. Patents—by design—short-circuit competition by giving the owners the legal right to prevent others from using (or even developing) their invention. TDI, on the other hand, would restore competition by making drug candidates available to anyone who wanted to develop them. We expect sponsors to exploit this advantage by signing development contracts with whichever company offers the lowest bid. Such competitive bidding is a powerful way to contain costs, and is also a good way to develop drugs. Virtual Pharma has extensive experience supervising contract research.

Third, the absence of patents would continue to keep prices low once drugs reached the market. The generic drug industry shows what happens once drug makers are allowed to compete. US drugs frequently fall to about one-third their original price when patents expire [12].

## Intellectual Property Rights

Would universities and corporations really let their people volunteer? Won't they insist on intellectual property rights? The practical answer is that sensible managers do not care about intellectual property rights unless they expect to earn a profit. This explains why sophisticated university licensing offices seldom bother to interfere with open-source software projects that are not commercially valuable [13]. The same logic would apply to open-source drug discovery. We would hope that life sciences companies would make a similar calculation. But permitting employees to participate is only the beginning. We think that universities and companies will also donate the data, research tools, and other resources needed to make TDI even stronger. The reason, once again, is that they have little to lose. The value of their intellectual property depends almost entirely on US and European diseases. For this reason, it costs very little to share their information with tropical disease researchers. In fact, drug companies already do this [14]. TDI's main challenge will be to show donors that an open-source project can keep members from diverting donated information back into the commercially lucrative diseases that affect patients in the West.

Finally, there are precedents for private companies developing drugs off patent. During the 1950s, March of Dimes (see www.marchofdimes.com) developed polio vaccines without any patents at all [15]. It then signed guaranteed purchase contracts with any drug maker willing to develop commercial-scale production methods. The incentive may not have been conventional, but it worked. And why not? The contracts made good business sense: contract profits may have been small compared to the profits on patented drugs, but so was the risk. Fifty years later, contract research still makes sense. Generic drug companies, developing world drug manufacturers, contract research organizations, and biotech firms have all said that they would consider contracts to develop open-source drug candidates. (M. Spino, S. Sharma, F. Hijek, and D. Francis, personal communications).

## Next Steps

So far, we have described a shoestring operation that exists mainly on the Web. Except for computer time, budgets would be more or less the same as existing software collaborations. Computing would be expensive but manageable. Today's biologists routinely scrounge resources from university machines or borrow time on home computers [16, 17]. This Web-centric approach would be a good start, but not a complete solution. Computational biology works best when it can interact with experimental chemistry and biology. Nevertheless, a low-budget computational approach is probably enough to generate new science, suggest ideas for follow-up experiments, and make new drug candidates available under licenses designed to yield maximum benefit to the developing world.

In practice, an open-source drug discovery effort is likely to include modest experiments. Many academic scientists control discretionary resources and, in some cases, tropical disease grants. Furthermore, good science generates its own funding. We expect experimentalists to turn the collaboration's Web pages into grant proposals.

That said, TDI's volunteers will be most productive if sponsors back them. Charities could support open-source drug discovery by making wet chemistry and biology experiments a top priority. Corporations could also help by donating funds, laboratory time, or previously unpublished results. One low cost/high value option would be for companies that have already tried a particular research direction to warn TDI if the collaboration was about to investigate a known dead end. (R. Altman, personal communication)

## Conclusion

Open-source drug discovery is feasible—that is, no known scientific or economic barrier bars the way. But what are the risks? Experience with software collaborations highlights the main social and economic challenges. First, the project will have to find and motivate volunteers. Based on existing software collaborations, we estimate a required minimum “critical mass” of a few dozen active members. Second, modest chemistry and biology experiments will be needed to increase the chances for success. Resources of several hundred thousand dollars per year—mostly in the form of in-kind donations of databases, laboratory access, and computing time—would make open-source drug discovery much more powerful. By most standards, such risks are real but acceptable.

The largest uncertainties are scientific. Can a volunteer effort based on computational biology and modest experiments produce the high-quality drug candidates that Virtual Pharma needs? A successful program must (1) make a significant contribution toward supplying the genomic insights that tropical disease research needs to move forward, and (2) make useful drug candidates available for development and production under open-source licenses. Open-source drug discovery looks feasible. The only way to be sure is to do the experiment—and we invite you to join us.

*To learn more about TDI or to volunteer, go to http://www.tropicaldisease.org*

## Abbreviations

R&D: research and development
TDI: Tropical Diseases Initiative
